# The Intersection of Socioeconomic and Environmental Factors in Aging: Insights from a Narrative Review

**DOI:** 10.3390/ijerph22081241

**Published:** 2025-08-08

**Authors:** Shelby Vereecke, Kalia Bennett, Stephanie Schrempft, Michael Kobor, Michael Brauer, Silvia Stringhini

**Affiliations:** 1 School of Population and Public Health, the University of British Columbia, Vancouver, BC V5T 1Z4, Canada; 2 Unit of Population Epidemiology, Division of Primary Care Medicine, Geneva University Hospitals, CH-1211 Genève 4 Geneva, Switzerland; 3 Edwin S.H. Leong Centre for Healthy Aging, The University of British Columbia, Vancouver, BC V5T 1Z4, Canada; 4 Department of Biochemistry and Molecular Biology, Faculty of Medicine, the University of British Columbia, Vancouver, BC V5T 1Z4, Canada; 5 Department of Health and Community Medicine, Faculty of Medicine, University of Geneva, CH-1211 Genève 4 Geneva, Switzerland

**Keywords:** aging, socioeconomic factors, environmental exposure, air pollution, health status disparities, social determinants of health, aged, adult

## Abstract

(1) Background: Socioeconomic conditions and environmental exposures are well-established determinants of health and aging, yet the pathways through which they influence the aging process remain insufficiently understood. Clarifying these mechanisms is critical for developing effective, equity-focused public health interventions to support healthy aging; (2) Methods: We conducted a narrative review examining the relationships between socioeconomic conditions, environmental exposures, and aging-related health outcomes. While the scope was intentionally broad to capture diverse exposures and outcomes, we applied a systematic search strategy to identify relevant peer-reviewed studies; (3) Results: The search populated over 4000 articles; 33 relevant papers were selected. The evidence suggests that environmental exposures may mediate or modify the effects of socioeconomic disadvantage on aging. Conversely, socioeconomic conditions can alter the association between environmental factors and aging outcomes. Disadvantaged populations consistently face higher environmental burdens and exhibit poorer aging outcomes, including accelerated biological aging and increased risk of age-related disease; (4) Conclusions: The complex interplay between social and environmental factors contributes to disparities in aging. Our integrative approach highlights the need for more intersectional, longitudinal research to inform interventions that address the social and environmental determinants of healthy aging.

## 1. Introduction

### 1.1. Background

In 2020, the number of people aged 60 or older officially outnumbered the population of children under five [1]. This demographic shift presents significant societal challenges, particularly as older adults may experience changes in health and functioning that impact their ability to fully engage in daily life activities and participate in their communities [2]. These challenges can be further compounded by ageism, which can isolate older individuals and strain intergenerational connections [2]. In Western societies, such isolation may be intensified by modern living arrangements, particularly the prevalence of nuclear households, where older adults are less likely to live with extended family [3]. Additionally, an aging population presents significant challenges to economic and healthcare systems [4]. A country’s economy may suffer from a shrinking labor force, with fewer working-age individuals contributing to economic productivity and tax revenues [4]. In addition, the increasing proportion of the population reaching old age increases demand for health services, further burdening healthcare systems that must address a growing number of chronic conditions among older adults [5]. The increasing direct and indirect healthcare costs further exacerbate financial strain, underscoring the urgent need for innovative strategies to support both economic stability and the evolving health needs of aging populations [1,4,5].

A growing body of research has highlighted the importance of social determinants of health and the physical environment in shaping functional outcomes in later life. However, these domains are often studied in isolation. The aim of this narrative review is to explore the interplay between socioeconomic conditions and environmental factors in relation to aging-related outcomes. By synthesizing findings across disciplines, this review draws attention to how these factors may interact, intersect, or compound to influence aging processes, with the goal of informing more integrated public health and policy responses.

#### 1.1.1. Aging: Functional Shift, Disease Incidence, and Trajectories of Life

Healthy aging is characterized by the maintenance of health and functional ability into older age [6]. For the purpose of the current review, functional decline, chronic disease incidence, quality of life, and life expectancy will be considered key aging-related outcomes [7]. The prevalence and pace at which these outcomes occur contribute to an individual’s aging trajectory [8]. Functional decline may occur in multiple areas, including physical, psychological, cognitive, sensory, and social health [7,9]. Physical decline may involve difficulties such as mobility, gait, strength, fine motor skills, coordination, and frailty [7,10]. Cognitive decline can affect memory, attention, concentration, and disorientation [9]. Sensory decline often includes hearing loss and vision problems, while social and psychological decline may manifest as isolation and depression [9,10]. Notably, declines in physical, cognitive, sensory, and psychosocial functioning can be interrelated and can influence one another bidirectionally throughout the aging process [9]. Aging-related diseases include, but are not limited to, dementia, type 2 diabetes, cardiovascular diseases, respiratory conditions, renal diseases, arthritis, osteoporosis, and certain cancers [11].

Ultimately, people experience aging differently, with some aging more quickly than others due to genetic makeup and the cumulative impact of various exposures and conditions [7]. This variability in aging can be captured through the concept of biological age, which reflects the physiological decline across various organ systems [12]. Biological age reflects aging at a cellular level; DNA methylation and some biomarkers are used to estimate epigenetic age, a specific measure of biological age [7]. Research indicates that individuals of the same chronological age can have significantly different biological ages, which serve as more accurate predictors of age-related diseases and mortality than chronological age [7,8]. The interplay between several factors, such as genetics, environment, lifestyle habits, and disease, contributes towards one’s biological age [8].

#### 1.1.2. Factors Influencing Aging Trajectories and Outcomes

Multiple factors contribute to health and aging trajectories, including sex, race, income, educational attainment, occupation, environmental exposures, behavioral risk factors, genetics, and early life experiences [8,13,14,15]. While researchers often explore the impact of socio-demographics and lifestyle factors on aging, the interplay between other factors remains understudied [16,17]. In particular, socioeconomic conditions, including income, occupation, and educational levels, as well as exposures to environmental factors, such as air pollution, green space, and endocrine-disrupting chemicals, have mostly been studied individually in relation to aging outcomes [16]. These existing studies frequently adjust for sociodemographic factors such as age, sex, and race to isolate the effects of socioeconomic conditions and environmental exposures [16]. However, research on how socioeconomic conditions and environmental exposures interact with one another and with sociodemographic factors (e.g., race, age, sex) in shaping aging trajectories remains limited [16,17]. Given the broad scope of both socioeconomic and environmental factors, the existing literature spans a wide range of contexts and variables, making it challenging to draw definitive conclusions [16]. Identifying the knowledge gaps within this body of research is crucial, as it can help direct future studies to explore the complex interplay between these determinants and their combined effects on aging [14,16].

#### 1.1.3. Socioeconomic Conditions and Aging

Socioeconomic conditions are major determinants of mortality, life expectancy, and aging [14]. For example, an individual’s education, occupation, or income may influence the rate at which functioning is lost [14]. Pearce et al. (2011) found that a 70-year-old from a high socioeconomic background may have an average physical health comparable to that of a 62-year-old from a lower socioeconomic background [18]. Similarly, Stringhini et al. (2018) found that, on average, a 60-year-old man of low socioeconomic status had the same walking speed as men aged 66.6 years with a high socioeconomic status [15]. This translates to an average loss of 6.6 years of functioning for men of low socioeconomic status [15].

Examining the indicators contributing to socioeconomic disadvantage is important for understanding how different socioeconomic conditions may have differential effects on aging, with the potential for cumulative impacts across the life course [13,19]. While the majority of studies focus on the role of income, other research has also explored the influence of factors like education, occupation, and wealth [20]. For instance, Macinko et al. (2023) followed over 9000 Brazilian adults and found that those with higher household income lived longer than those with lower household incomes [21]. In terms of education, researchers have found higher educational attainment to be associated with slower epigenetic aging [22], later menopausal onset [23], better lung function [24,25], and reduced incidence of disease, including hypertension [26]. As for occupation-related socioeconomic conditions, Iavicoli & Cesari (2018) performed a systematic search that supported the finding that men working manual or blue-collar jobs were more likely to experience frailty in older age [20]. Lastly, wealth has been measured in certain longitudinal studies of aging, such as the English Longitudinal Study of Aging [27]. Using this data, Torres et al. (2016) found less wealth, which is the accumulation of assets over the life course, to be associated with greater disability compared to wealthy older adults [27]. These studies make up only a small number of articles in the body of literature evaluating the association between socioeconomic conditions and aging. As seen within the literature, the impact of socioeconomic conditions on aging is apparent. While the current review focuses on individual-level socioeconomic conditions (e.g., occupation, income, education), it is notable that community-level deprivation (e.g., proximity to care, neighborhood safety) may also pose risks to aging outcomes [28].

#### 1.1.4. Environmental Exposures and Aging

The environmental exposures impacting aging trajectories and outcomes are extensive [29]. To contextualize these exposures, this review adopts Bronfenbrenner’s socioecological model [30,31]. This model categorizes exposures, from immediate settings such as the household level (micro-level) to broader influences like neighborhood greenness (typically exo-level) and societal norms or policies at the country level (macro-level) [30,31]. For the purpose of this review, neighborhood-level exposures will be considered macro-level to streamline result analysis while discussing environmental exposures at both the micro- and macro-levels [29]. While the following exposures are the most commonly researched, they represent only a subset of environmental exposures linked to aging in the literature [29].

*Macro-level*. Environmental exposures occurring on a larger scale include air, soil, light, and noise pollution [29,32,33]. Of such factors, air pollution has been the most extensively researched [32]. Commonly researched polluting particles include fine particulate matter (PM 2.5), which is a mixture of air particles that are 2.5 μm or less in size [29,32,34,35]. The small size of these particles allows for them to be inhaled deeply into the lungs [32].

Research investigating the link between environmental exposures, in particular air pollution, and aging-related conditions like dementia and cancer is becoming more common. For instance, a cohort study conducted in London, England, found an association between the exposure to PM 2.5 and higher dementia incidence in older adults [36]. Similarly, an Australian longitudinal study found PM 2.5 exposure to be associated with more new cases of vascular dementia [37]. Researchers have frequently linked air pollution exposure to lung cancer risk; however, the impact of air pollutants on other forms of cancer is less studied [38,39]. To date, nitrogen dioxide exposure has been linked to increased risk of breast cancer [40], and PM 2.5 has been shown to increase the risk of liver cancer [41].

Research on other macro-level environmental factors like noise, water, and soil exposure is more limited [29,33,42]. In terms of noise pollution, a review of the literature highlighted an abundance of articles supporting the relationship between noise exposure (e.g., railroad noise, aircraft noise, traffic-related noise) and cardiovascular health outcomes [33]. In particular, elevated noise exposure was found to be associated with higher systolic blood pressure and more cases of atherosclerosis [43,44,45]. In more serious cases, studies reported a higher incidence of stroke in relation to loud noise exposure [46,47]. Moreover, a recent review article covered the impact of heavy metals found in water and soil across different countries [42]. The article highlighted the cardiotoxic effect these pollutants are having on human health, leading to an increase in cardiovascular disease risk, which is a well-known aging-related disease [42].

In terms of the link between macro-level exposures and healthy aging (e.g., functional outcomes and biological age), this emerging area of research is still rather limited [29]. From the existing body of literature, a German study found elevated air pollution exposure to be associated with older epigenetic age, based on blood samples from 1799 older adults [48]. Further, Keidel et al. (2019) found that greater exposure to traffic-related pollution was associated with poorer lung function among older adults [24]. Other studies found PM 2.5 exposure to be associated with increased frailty and mortality in older adults [29,34,35].

Some macro-level exposures may play a protective role in aging [49]. Multiple studies have shown that good access to walkable, green, and blue spaces can improve aging outcomes [50,51,52]. For example, studies across various countries have reported results supporting the association between longevity and walkable, blue and green spaces [49,50,53,54]. More specifically, an English qualitative study investigated the effect of coastal blue space on well-being in adults over 50 [55]. In this study, participants with access to blue and green spaces reported social connectedness and good physical and mental health [55]. Ultimately, exploring both adverse and protective factors can better inform environmental policy interventions that promote healthy aging [29,55].

*Micro-level.* Individual-level environmental exposures related to everyday items such as food, cosmetics, furniture, and housing materials significantly influence aging trajectories [56]. Food consumption can lead to exposure to pesticides and heavy metals, such as mercury, cadmium, and arsenic, which accumulate in the body over time and are linked to chronic health problems and accelerated aging processes [42]. Additionally, endocrine-disrupting chemicals, such as bisphenol A (BPA) and phthalates, are frequently found in food packaging, household plastics, and certain cosmetic products, while flame retardants in furniture and bedding can release harmful compounds into indoor environments [57]. Housing-related hazards, such as mold, dampness, lead, and copper contamination in drinking water, can exacerbate chronic inflammation and other biological processes associated with accelerated aging [42]. Although less apparent in daily life, these exposures are pervasive and can lead to long-term health effects that contribute to disparities in aging outcomes [42,56,57].

In low- and middle-income countries, individual-level environmental exposures often include additional risks linked to polluting cooking fuels such as gas, coal, wood, or agricultural residues [56]. A study conducted across six countries (China, Russia, Ghana, India, Mexico, and South Africa) found that individuals relying on these fuels for cooking and heating had significantly higher odds of developing arthritis compared to those using electricity [56]. Additionally, a systematic analysis from the 1990–2021 Global Burden of Disease (GBD) study estimated exposure to household air pollution exposure from cooking fuels [57]. GBD collaborators found that one-third of the global population used polluting cooking fuels, and in 2021, household air pollution was responsible for an estimated 111 million global disability-adjusted life years [57]. While these exposures are less common in high-income settings, the findings highlight the global disparities and the broader relevance of micro-level environmental exposures [56].

Beyond air pollution, endocrine-disrupting chemicals, such as BPA and phthalates, have been associated with premature ovarian aging in peri- and post-menopausal women, as shown in a 2024 study [58]. Together, these findings underline the importance of addressing individual-level exposures across contexts, emphasizing their role in driving health inequities and aging disparities.

#### 1.1.5. Social Patterning of Environmental Exposures

The literature indicates that environmental exposures may be unevenly distributed across different socioeconomic groups [59,60]. For instance, a comprehensive review found that most North American studies reported elevated air pollution levels in socioeconomically disadvantaged areas [61]. Outside of North America, cities in New Zealand, Italy, and Hong Kong have observed similar trends [61,62]. In contrast to these findings, recent studies in Canada examined the relationship between socioeconomic status and exposure to air pollution, yielding mixed results [60,61]. A 2016 study by Statistics Canada analyzed nitrogen dioxide (NO_2_) exposure among children in Toronto, Montreal, and Vancouver [60]. The findings indicated that in Toronto and Vancouver, children from lower-income households experienced higher NO_2_ exposure levels [60]. Conversely, in Montreal, children from higher-income households were more exposed to NO_2_ [60].

As for noise pollution, a Canadian study mapped exposure levels across Montreal and found noise pollution to be increased in areas of lower socioeconomic position [59]. Like air pollution, the distribution of noise pollution varies depending on the global context [33]. Germany, Hong Kong, and most cities in the USA reported similar noise pollution trends to Canada [59]. However, in cities like New York, adults of higher socioeconomic position are more likely to live in areas with high levels of traffic-related noise [33]. Therefore, it is important to consider that the social and geographical patterning of environmental exposures may vary between cities and regions [33].

At the individual level, frequent exposures to BPA and other endocrine disruptors have been unequally experienced by low-income individuals. Two American studies, by Ruiz et al. (2018) and Nelson et al. (2012), found BPA, phthalates, and indoor air pollution to be associated with food insecurity, low household income, and having received emergency food assistance [63,64]. These studies also found Mexican American and African American participants to be disproportionately exposed to these environmental exposures compared to participants of other racial groups [63,64].

In brief, the social patterns explaining the distribution of environmental exposures are complex, and therefore, an effective research approach must consider age, culture, race, income, education, and environment [33,59,63].

#### 1.1.6. Hypothesis

Exposure to poor socioeconomic conditions may increase exposure to environmental hazards, such as air pollution, lead, mold, BPA, and proximity to industrial areas [65,66], which in turn accelerates biological aging [15,58]. Despite low socioeconomic status being associated with a plethora of suboptimal environmental conditions, the literature exploring the interplay between these factors in relation to aging outcomes is rather recent and sparse.

This narrative review is based on the hypothesis that socioeconomic conditions and environmental exposures intersect to influence aging trajectories, rather than acting independently. Two potential pathways are considered: first, that environmental factors mediate the link between socioeconomic conditions and aging, and second, that environmental and socioeconomic factors interact to predict aging-related outcomes. We hypothesize that both pathways (mediation and effect modification) are represented in the literature, suggesting that both may be valid in explaining the relationship between socioeconomic conditions, environmental exposures, and aging. By examining the existing literature, this review aims to demonstrate the interconnected nature of these factors and their combined impact on the biological mechanisms of aging.

### 1.2. Objective

The aim of this narrative review is to explore the interplay between socioeconomic conditions and environmental factors in relation to aging-related outcomes. Specifically, this review seeks to address the following research questions:

1. What is the current evidence on the interplay between environmental factors, socioeconomic conditions, and aging outcomes?
2. What key knowledge gaps exist within this area of research?

## 2. Methods

### 2.1. Eligibility Criteria

The objective of the current narrative review was to identify research exploring the interplay between socioeconomic conditions and environmental factors in relation to aging outcomes. The selection of inclusion and exclusion criteria is highlighted in Table 1 and followed the PICO format, which stands for population, intervention/exposure, comparison, and outcome [67].

Studies were considered for review if they met the following inclusion criteria: (1) The study was published in English; (2) The study investigated the interplay between socioeconomic and environmental exposures in relation to aging outcomes in human participants; (3) The study was published no earlier than 2014; (4) The study population included adults or older adults. Longitudinal studies including participants under the age of 18 were included if outcomes were associated with adult-related aging outcomes. Studies investigating aging outcomes in neonates or children (e.g., biological age, developmental delays) were excluded from this review in order to narrow the scope.

### 2.2. Search Strategy

Articles studying the socioeconomic-environmental exposure interplay and aging outcomes were searched. The literature search was performed on MEDLINE and Scopus, including only full-length studies involving human participants published in the last 10 years.

The scope for this search was intentionally broad, with three key themes included in the search string: (1) Socioeconomic Conditions, (2) Macro- and Micro-Level Environmental Factors, and (3) Aging Outcomes. The full search string can be found in Appendix A**.** Medical Subject Headings (MeSHs) terms were incorporated where appropriate. Quotation marks were included to search for common phrases (e.g., Lead Poison), where words on their own would overexpand the scope of the search (e.g., Lead).

The search strategy was refined by a reference librarian specialized in public health (UE). The authors also used OpenAI’s ChatGPT (version GPT-4-turbo) to assist in identifying key terms for the search strategy; the output was carefully reviewed, and the authors take full responsibility for the search and the content of this manuscript. To optimize the identification of studies, the search strategy was revised by a second (KB) and third reviewer (SS). The search strategy was run in two databases initially in October 2024 and again in March 2025 to comprehensively capture relevant articles.

### 2.3. Selection Process

**Figure 1:**
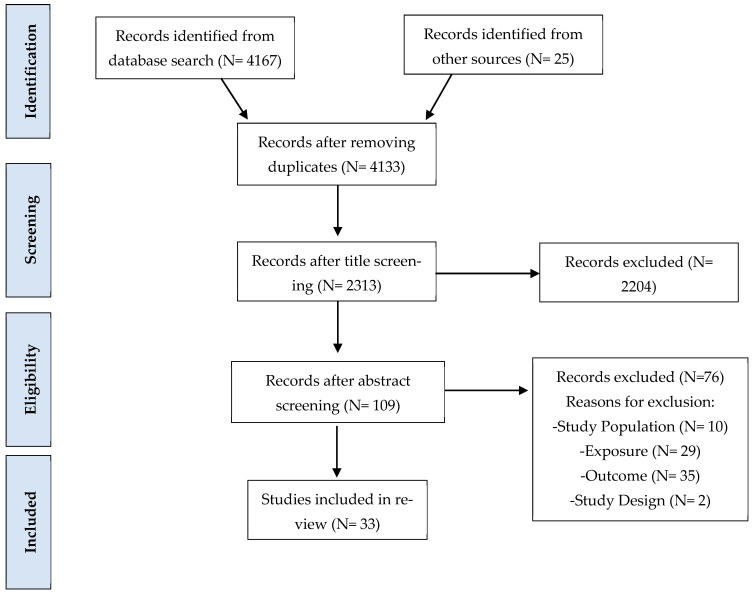
PRISMA flowchart depicting the methods of the literature review.

Covidence Systematic Review Software was used to assist in selecting studies for the literature review [68]. Using this software, duplicates were removed, and the titles and abstracts of the remaining studies were reviewed to identify those that possibly met the inclusion criteria [68]. For each study that was deemed relevant after the title and abstract screening, the full-text version was screened thoroughly by the primary reviewer (SV). The selection process was reviewed in Covidence Systematic Review Software (KB). Each study was marked as either included or excluded after review. All excluded studies were individually labelled with reasons for exclusion. The initial search yielded over 4000 studies, which were screened for eligibility and led to the inclusion of 33 studies in this review (Figure 1).

### 2.4. Data Extraction

All relevant information for each study was imported into a Microsoft Excel spreadsheet by the primary reviewer (SV). Characteristics of the study (e.g., study location, date), sample (e.g., target population, recruitment strategy, mean age of participants), exposures (e.g., socioeconomic conditions and environmental factors), outcome (aging), and statistical analysis (e.g., causal mediation analysis, regression analysis) were included in the data that was extracted.

## 3. Results

### 3.1. Review of the Literature

A complete summary of the included articles can be found in Table 2. Of the 33 included articles, eleven studies were conducted in North America, two studies in South America, nine studies in Europe, five studies in Asia, three studies in Oceania, and three of the studies were carried out across multiple continents. Most of the empirical studies were longitudinal (N = 18), with the remaining 13 following a cross-sectional design. Two of the papers included were systematic review articles.

Each of the included articles investigated how the interplay between socioeconomic conditions and environmental factors may be associated with aging. In terms of socioeconomic conditions, the articles included in this review focused on income, education, occupation, house value, and employment status. As for environmental factors, most studies evaluated macro-level exposures, such as green space access, PM_2.5_, and ozone (O_3_) (N = 29). Other studies investigated household exposures, such as polluting cooking fuels or endocrine-disrupting chemicals (N = 4).

Lastly, various aging outcomes were explored in relation to the interplay between socioeconomic and environmental exposures. These outcomes include self-rated health and quality of life (N = 4), functional outcomes (e.g., walking speed [N = 1], lung function [N = 4], cognitive function [N = 6]), aging-related conditions (e.g., diabetes [N = 2], cardiovascular conditions [N = 6], hypertension [N = 1], arthritis [N = 1], osteoporosis [N = 1], dementia [N = 3]), health indicators (N = 1, e.g., systolic blood pressure, C-reactive protein levels, body mass index), ovarian aging and menopause (N = 2), aging trajectories and acceleration (N = 3), and life expectancy and mortality (N = 3).

### 3.2. The Interplay Between Socioeconomic Conditions and Environmental Factors

The majority of studies identified in this review explored the effect of socioeconomic conditions on the association between environmental exposures and aging outcomes. Fourteen studies examined whether the environment-aging association held after adjustment for socioeconomic conditions, and eight of the studies evaluated the modifying effect of socioeconomic conditions on the environment-aging association. As for the association between socioeconomic conditions and aging, the mediating (N = 4) and modifying effect (N = 3) of environmental exposures were explored.

#### 3.2.1. Associations Between Environmental Exposures and Aging: The Role of Socioeconomic Conditions

Studies from the literature have explored both the hypothesized confounding and modifying effect of socioeconomic conditions in terms of the association between environmental exposures and aging outcomes.

*Hypothesized confounding effect*. This review included fourteen studies adjusting for socioeconomic conditions in the association between environmental exposures and aging outcomes.

For instance, three studies investigated the association between air pollutant exposure and dementia incidence, adjusting for socioeconomic conditions (e.g., income, education, occupation). First, Carey et al. (2018) found NO_2_ to be associated with increased dementia incidence when participants were exposed to a minimum of 41.5 µg/m^3^ annually, which could not be explained by confounding [36]. Contrastingly, a Canadian study investigated this same association and found that higher income and education attenuated the impact of air pollution exposure on dementia incidence [70]. Similarly, Trevenen et al. (2022) found PM_2.5_ to be associated with vascular dementia incidence; however, once adjusting for socioeconomic conditions, this association was attenuated [37].

While the fourteen included studies investigated different environment-aging associations, most studies found that income, education, and/or occupation confounded the association between environmental exposures and aging outcomes. Contrarily, some studies found the environment-aging association to be unaffected by socioeconomic conditions. For example, White et al. (2019) explored the association between air pollution exposure and epigenetic age acceleration, adjusting for socioeconomic conditions [75]. They found that NO_2_ was inversely associated with epigenetic age acceleration, and socioeconomic conditions did not notably confound the association [75].

Ultimately, the majority of studies included in this review found the environment-aging association held after adjustment for socioeconomic conditions. The full outline of findings can be found in Section A of Table 2.

*Effect modification.* In terms of socioeconomic conditions acting as effect modifiers in the association between environment and aging, the assumption is that the effect of environmental factors on aging outcomes may depend on whether individuals are also exposed to adverse socioeconomic conditions. The potential modifying effect of socioeconomic conditions was examined by eight articles included in this review. As seen in Section B of Table 2, the findings of the selected studies varied a great deal.

To start, four of the eight selected articles found socioeconomic conditions to modify the association between environmental exposures and aging outcomes. A recent study by Cui et al. (2024) found PM 2.5 exposure had a stronger impact on cardiovascular outcomes in participants of low SES residing in poorer regions [32]. Another recent Chinese study investigated the impact of built environment accessibility on the pace of cognitive decline, with educational attainment (high/low) being considered as an effect modifier [82]. They found that among participants with limited access to built environments, higher-educated participants exhibited a slower decline in cognition compared to low-educated participants [82]. Moreover, in the United States, Kim et al. (2023) analyzed longitudinal data with the aim of exploring the modifying effect of neighborhood deprivation on the association between green space access and biological age (GrimAge acceleration) [79]. They found neighborhood deprivation to intensify the association between greenness and slower epigenetic aging [79]. Another American longitudinal study found neighborhood socioeconomic disadvantage to modify the relationship between PM_2.5_ and self-rated health among older adults [80]. However, this study found the association between PM_2.5_ and self-rated health to be exacerbated in higher-income neighborhoods [80]. On the other hand, the association was weaker in lower-income neighborhoods [80].

Furthermore, two articles found that socioeconomic conditions did not act as effect modifiers when tested in their analyses. A 20-year longitudinal study conducted across multiple European cities investigated the impact of green spaces on the age of menopause, stratifying by educational attainment [23]. These researchers found that while women with less green space access became menopausal on average 1.4 years sooner than women with good green space access, educational attainment did not modify this association [23]. Also, Heo et al. (2022) investigated the effect of air pollution exposure on osteoporosis-related fracture risk [77]. Likewise, they found that income level did not modify this association [77].

The remaining two articles reported mixed findings related to the modifying effect of socioeconomic conditions. A case-crossover study found those exposed to poor socioeconomic conditions to have higher prevalence of cardiac arrests when exposed to low-medium levels of wildfire smoke [78]. However, when wildfire smoke exposures were high, cardiac arrest prevalence did not differ between low and high socioeconomic groups [78]. Finally, Qiu et al. (2022) explored the relationship between O_3_, PM_2.5_, and NO_2_ exposure and age-related psychiatric symptoms in a longitudinal study [81]. This study found income and house value to modify the association between O_3_ and psychiatric symptom prevalence; however, the same was not found for the other air pollutants [81].

#### 3.2.2. Associations Between Socioeconomic Conditions and Aging: The Role of Environmental Exposures

When exploring the effect of socioeconomic conditions on aging outcomes, recent literature presents mixed findings on the role of environmental exposures.

*Effect modification*. The potential modifying effect of environmental factors was presented by three studies in this review. These studies explore whether the impact of socioeconomic conditions on aging outcomes depended on the exposure to environmental factors.

For instance, Mitchell et al. (2015) investigated the modifying effect of environment on the association between financial strain and mental health, incorporating proximity to green spaces as an interaction term [51]. Their findings indicated that the negative impact of poor socioeconomic conditions on mental health outcomes was less pronounced for those with good access to green areas [51].

In another cross-sectional study, Koh et al. (2022) explored the modifying effect of green space accessibility on the association between socioeconomic conditions and hypertension outcomes [26]. While a dose-response relationship was found between educational attainment and hypertension, green space accessibility did not serve as an effect modifier in this study [26].

In a longitudinal study, Vilarino-Rico et al. (2023) stratified their analysis by urban and rural living environments, examining the relationship between socioeconomic disadvantage and major adverse cardiovascular events (MACEs) [83]. Interestingly, participants facing socioeconomic disadvantage living in rural environments were neither more nor less likely to experience MACE compared to other participants [83].

Notably, the latter two studies found environmental exposures (green space access and population density) to not act as significant effect modifiers [26,83], while the first study presented found green space access to show significant interaction with financial strain in terms of mental health outcomes [51]. While each of these studies explored different research questions, the findings showcase that the potential modifying effect of environmental exposures is unclear.

*Mediation.* Four articles included in the review explored whether environmental exposures played a mediating role between socioeconomic conditions and aging outcomes.

Three of these studies investigated the potential mediating effect of air pollution. A recent cross-sectional study explored the possible mediating role of NO_2_ and PM_10_ exposure in the association between educational attainment and lung function using a causal mediation framework [25]. As a result, these researchers concluded that higher educational attainment served as a protective factor against age-related lung function decline, with 12% of the association being mediated by air pollutant exposure (PM_10_ and NO_2_) [25].

In the second study, Chaparro et al. (2018) explored the potential mediating effects of SO_2_, PM_10_, NO_2_, and CO in the association between socioeconomic deprivation and common health indicators (lung function, BMI, systolic blood pressure, and C-reactive protein levels) [85]. This longitudinal study found that only SO_2_ partly mediated the relationship between socioeconomic deprivation and health indicators, with the exception of lung function, which was not associated with socioeconomic deprivation [85].

Next, in an American study, Canterbury et al. (2020) analyzed whether the impact of social risk (e.g., racial minority, single living, low income, and low educational status) on cardiovascular disease (CVD) risk was mediated by 1-year PM_2.5_ exposure [84]. As a result, CVD risk was found to be higher among those with increased social risk, and 13% of this association was explained by PM_2.5_ exposure [84].

The fourth study presented in this review analyzed the possible mediating effect of green space access on the relationship between socioeconomic conditions (education, income, occupation) and the incidence of type 2 diabetes [16]. While higher type 2 diabetes incidence was related to worse socioeconomic conditions, green space access did not prove to act as a strong mediator in this association [16].

## 4. Discussion

### 4.1. Overview of Findings

The current narrative review assessed the existing body of literature investigating the interplay between socioeconomic conditions, environmental factors, and aging outcomes. Resultingly, 33 relevant articles were selected for inclusion.

#### 4.1.1. The Scope of the Review

The current narrative review applied a broad scope with the aim of highlighting the existing evidence on the interplay between environmental factors, socioeconomic conditions, and aging outcomes. The literature search was completed systematically to capture articles studying various environmental exposures (e.g., air pollution, population density, neighbourhood security, walkability, green and blue space access, etc.), socioeconomic conditions (e.g., income, education, occupation, employment status, home ownership, etc.), and aging outcomes (e.g., walking speed, disease incidence, life expectancy, quality of life, epigenetic aging, etc.).

Additionally, other review articles exploring this interplay were identified in the current search. However, unlike this narrative review, existing review papers applied a narrower scope. For instance, Ruiz et al. (2018) reviewed the literature investigating the impact of environmental exposures, including BPA, phthalates, and air pollution, on diabetes incidence while controlling for variables, such as household income and race [63]. This review paper found that most studies reported pollution exposure to be associated with increased diabetes risk and worse outcomes among those with lower income [63]. Like other review articles, Ruiz et al. (2018) focused on papers that highlighted specific environmental exposures, socioeconomic conditions, and a single aging-related outcome, such as type 2 diabetes [63]. To the authors’ knowledge, the current narrative review is one of the first articles to apply a broad scope to a systematic search of the literature, which investigated the interplay between socioeconomic conditions, environmental exposures, and aging outcomes.

#### 4.1.2. Overview of the Socioeconomic-Environment-Aging Interplay

Most of the included studies covered how socioeconomic conditions may modify or explain the relationship between environmental factors and health outcomes [24]. Notably, higher education and income attenuated the harmful effects of environmental exposures on aging outcomes in nearly all relevant articles [70]. As for assessing for effect modification, the eight studies evaluating the modifying effect of socioeconomic conditions reported conflicting results. Four articles found socioeconomic conditions to modify the association, two studies found the environment-aging association did not depend on socioeconomic conditions, and the remaining two articles found that the modifying effect of socioeconomic conditions was contingent upon environmental exposure levels being high. Considering the mixed results reported by the selected articles, which evaluated various contexts, the role of socioeconomic conditions in the relationship between environment and aging appears to be complex and context-dependent [24]. Interestingly, Keidel et al. (2019) investigated the association between NO_2_ exposure and lung function [24]. This study tested whether socioeconomic conditions modified or confounded the association under investigation [24]. They found that socioeconomic conditions did not modify the relationship, and of all the socioeconomic conditions tested in the models, only education confounded the NO_2_-lung function association [24]. These researchers speculated that the interplay between education and NO_2_ exposure was mediated by other lifestyle factors, such as smoking status and BMI [24]. Keidel et al. (2019) concluded that the interplay between socioeconomic conditions and environmental exposures must be investigated in order to improve model fit and further understand the cumulative impact on aging outcomes, such as lung function [24]. The authors of the current narrative review speculate that socioeconomic conditions contribute more substantially to the environment-aging association than would be expected if they were merely acting as confounders. However, given the heterogeneity in exposures and outcomes examined across studies, this relationship warrants further evaluation.

As for the role of environment, seven studies included in the review discussed how pollution and neighborhood quality may modify or mediate the effect of socioeconomic conditions on aging outcomes. Mitchell et al. (2015) revealed that access to green spaces may mitigate some negative health outcomes associated with socioeconomic disadvantage [51]. However, other included studies investigating the modifying role of the environment found that the association between socioeconomic conditions and aging was unchanged by the environmental factors assessed [26,83]. Similarly, when applying a causal mediation framework to assess the role of the environment on the socioeconomic-aging association, studies reported inconsistent results. Based on the suggested findings, the evidence supporting the mediating role of environment is sparse and requires further investigation.

Overall, the findings illustrate that both socioeconomic and environmental factors play crucial roles in shaping health outcomes during aging. The current review showcased some of the remaining knowledge gaps that exist on this subject. However, it is notable that with the body of literature being spread across various contexts, the evidence is limited for each environmental exposure and socioeconomic condition covered in this review.

### 4.2. Gaps in the Literature

While exploring the interplay between socioeconomic conditions and environmental exposures, most studies found a relationship between these two exposures and aging outcomes. However, the methodology and scope applied varied between studies, leaving gaps in the literature.

#### 4.2.1. The Role of Environmental Exposures

Most studies have focused on the confounding or modifying effects of socioeconomic conditions, while fewer have explored potential mediating and modifying effects of environmental exposures. One such study by Quispe-Haro et al. (2024) suggests that environmental factors may partially mediate the relationship between socioeconomic conditions and aging [25]. This study, one of the first to examine the mediating effect of air pollution on the link between education and lung function, highlights a gap in the literature [25]. There is still much room for future research to investigate how other environmental exposures, such as household pollutants, noise pollution, and access to green spaces, might mediate the relationship between various socioeconomic conditions (e.g., income) and aging outcomes, including functional decline or disease risk [24].

Moreover, although this search highlighted many longitudinal studies, most studies measured environmental exposures cross-sectionally. This is particularly important when studying exposures like lead, noise pollution, and air pollution, where long-term exposure to low levels can be as impactful as short-term exposure to high concentrations [86,87,88].

Finally, the complex interactions between many environmental exposures in relation to aging should be explored [25,36]. While this review has highlighted studies focusing on specific environmental factors and their impact on aging, a more comprehensive analysis of these exposures—considering their cumulative and synergistic effects—would offer an improved understanding and better reflect the everyday environmental exposures individuals face [36,69].

#### 4.2.2. Aging Outcomes: Socioeconomic and Environmental Interactions

Various aging outcomes were explored in relation to socioeconomic conditions and environmental factors as separate exposures. However, when examining the interplay associated between exposures, many aging outcomes were not extensively researched across multiple studies. For example, biological age, grip strength, frailty, walking speed, and bone mass deterioration are key indicators of aging that have all been linked to socioeconomic conditions and environmental exposures individually [12,15,34,89,90]. There is still an opportunity for researchers to explore these key functional indicators of aging in relation to both socioeconomic conditions and environmental factors simultaneously.

#### 4.2.3. Scope of Measures

Most studies investigating the interplay between environmental factors, socioeconomic conditions, and aging take a macro-level approach, often comparing multiple neighborhoods, countries, or continents. As an example, multiple articles have analyzed neighborhood-level air pollution exposure and income [91]. Fewer studies, however, have focused on individual-level exposures, such as personal income and household environmental exposures like lead and mold [29,92]. These daily exposures are often difficult to measure, leading to underrepresentation in the literature. A notable study from Western Europe found that the association between socioeconomic conditions and the level of environmental exposure varied depending on whether personal (e.g., income, assets) or neighbourhood-level (e.g., neighbourhood socioeconomic strata, average home price) socioeconomic conditions were assessed [66]. This highlights a critical gap in understanding how micro-level environmental exposures interact with socioeconomic position and their combined impact on aging outcomes [66].

#### 4.2.4. Global Representativeness

A notable gap in the literature is the lack of global representation among the studies included in this review. Few studies have examined the intersection of socioeconomic conditions and environmental exposures in relation to aging outcomes, particularly in low- and middle-income countries. This gap limits our understanding of how these factions may interact across different cultural, political, and infrastructural contexts. While Yamamoto et al. (2019) drew on multinational data including cohorts from China, Russia, Ghana, India, Mexico, and South Africa, the majority of studies came from high-income countries [56]. This imbalance reflects a broader need for more research that explores how environmental and social determinants jointly shape aging in underrepresented regions. Moreover, cross-country variation in how aging, health outcomes, and exposures are conceptualized and measured must be considered when interpreting and applying findings across contexts.

### 4.3. Limitations of the Search

The broad scope of the search strategy aimed to encompass a wide range of exposures and aging outcomes, but this approach may have resulted in the omission of relevant articles. Specifying types of exposures and health outcomes could have further narrowed the results and provided more focused insights. However, considering the intention was to conduct a narrative review to explore the existing general knowledge surrounding the interplay between environmental and socioeconomic exposures and aging, the chosen scope was appropriate for the objectives of this paper. Additionally, there is a risk of bias regarding the interpretation and selection of articles. However, the search strategy was reviewed by other authors to mitigate some of this bias.

### 4.4. Future Directions

Future research should focus on exploring both micro- and macro-level exposures in terms of socioeconomic conditions and environmental factors. This approach could reveal the intersectional nature of the relationship between personal socioeconomic status, neighborhood- and country-level wealth, household hazards, environmental exposures, and aging outcomes.

Further, research should continue to explore the biological mechanisms underlying the connection between the socioeconomic-environment interplay and aging outcomes. Understanding how these exposures impact functional and cognitive decline, as well as disease risk in aging populations, could enable tailored supports and targeted interventions.

In relation to the socioeconomic-environment interplay, there was a limited number of studies applying mediation analyses. The included studies that employed such methods varied in study populations, which is important when considering air pollution exposure and wealth. This gap warrants future cross-comparative research.

Lastly, the body of literature would benefit from additional longitudinal studies, as only a few relevant studies with this design were identified in the search. Outcomes in aging, such as the speed at which people experience functional decline, carry out over multiple years. Cross-sectional analyses are limited in capturing aging trajectories, disease incidence, and health outcomes over time.

### 4.5. Conclusions

This narrative review has explored the intricate relationship between socioeconomic conditions, environmental factors, and health outcomes related to aging. The findings indicated that poorer socioeconomic conditions were associated with accelerated aging [22], reduced walking speed [15], and earlier mortality [93], while environmental exposures such as air pollution may increase epigenetic age [48], decrease lung function [24], and negatively impact cognitive health [36].

Despite the growing body of literature, the remaining knowledge gaps impede our understanding of the underlying mechanisms that connect socioeconomic conditions and environmental factors to aging outcomes. Future research should delve deeper into the interplay between these exposures, particularly the potential mediating effect of environmental hazards on the relationship between socioeconomic conditions and aging trajectories. Furthermore, the protective role of a built environment, including green and walkable spaces, should be increasingly explored to understand how accessibility to green and walkable spaces may mitigate health risk [51]. By addressing these gaps, the development of targeted interventions to promote healthy aging trajectories can be facilitated [14]. Ultimately, understanding the interplay between environmental and socioeconomic factors is essential to advancing the global conversation on environmental justice and equitable aging [18].

## Figures and Tables

**Table 1:** Defining PICO criteria.

Criteria	Definition
Population	Adults
Exposures	Environmental exposures: Macro-level (Air pollution (e.g., NO_2_, PM_10_, PM_2.5_, SO_2_, O_3_) and community factors (e.g., green space, blue space, walkability, noise pollution, light pollution)) and micro-level (Household factors (e.g., mold, lead, endocrine-disrupting chemicals, heating, water, leaking, infestations)); Socioeconomic conditions: Income, education, wealth, housing, food security, social class, neighborhood deprivation, and occupation.
Comparison	Adults experiencing different socioeconomic conditions and/or environmental exposures
Outcomes	Aging (e.g., epigenetic age, biological markers, frailty, longevity, life expectancy, mortality), physical health (e.g., bone density), cognitive health (e.g., memory, cognitive function), functioning (e.g., walking speed, balance, grip strength, lung function), and age-related diseases (e.g., dementia, cardiovascular disease, respiratory diseases, cancer, diabetes)
Study Designs	Empirical quantitative and qualitative studies Review articles were also included for contextualization

**Table 2:** Summary of studies examining the interplay between socioeconomic and environmental factors related to aging outcomes (N = 33).

Study	Country	Design	Sample	Exposure	Outcomes	Major Findings
Macinko et al., 2023 [21]	Brazil	Longitudinal	9412 adults aged 50+	Sociodemographic variables, socioeconomic conditions (income, occupation, home ownership), smoking status, physical environment (urban vs. rural), social support (partnered vs. single), health (self-rated health, memory), and functional abilities (activities of daily living and grip strength)	Life expectancy	High school completion, social support (partnered), and female sex were negatively associated with mortality; Higher household income was more prevalent among those that survived the length of the study; Those that were loss to follow-up were less likely to have owned a home; Life expectancy did not vary based on physical environment.
Motoc et al., 2023 [69]	Netherlands	Longitudinal	2165 adults (55–85 years old)	Urban density, income, safety, proximity to retail, access to green spaces, water coverage, pollution (PM_2.5_), traffic noise, housing quality	Cognitive health, depression, and anxiety incidence	Anxiety incidence was associated with higher urban density, greater proximity to retail facilities, lower housing safety and quality scores, less access to green spaces, and higher PM_2.5_ levels.
Stephens et al., 2018a [14]	New Zealand	Longitudinal	13,040 adults (55–70 years old)	Sense of financial security, social support, housing quality, social cohesion, and neighborhood safety, accessibility, and walkability	Aging trajectories: physical, mental, and social	Using latent profile growth analysis, the five aging trajectories defined health as “robust”, “average”, “declining physical health”, “limitations in mental health and social well-being”, or “vulnerable”; Participants in the “robust health” aging group scored significantly higher in terms of financial security, housing quality (e.g., keeping home warm), neighborhood quality (e.g., safety, walkability), and social cohesion.
Stephens et al., 2018 [19]	New Zealand	Cross-sectional	3036 adults (50–89 years old)	Chronic conditions, environment (urban density and accessibility), socio-economic status, and housing (neighbourhood safety, social cohesion, financial security, accessibility, and walkability)	Perceived quality of life	Female gender, higher socioeconomic status, and fewer chronic conditions were linked to higher quality of life; Urban density modified the association between financial security and quality of life. Being from rural areas intensified this association.
Section A: Association between environmental factors and aging outcomes—with adjustment for socioeconomic conditions						
Carey et al., 2018 [36]	England	Cross-sectional	130,000 adults (50–79 years old)	Exposure: Traffic noise and air pollution (NO_2_, PM_2.5_, O_3_) Covariates: Area deprivation, age, sex, race	Dementia incidence	Night noise was associated with NO_2_ and PM_2.5_ exposure; All pollutant levels decreased with increasing distance from major roads; For those with an annual NO_2_ exposure of more than 41.5 µg/m^3^, an increase in dementia incidence was observed. The same was not found for those with lower levels of NO_2_ exposure; The association between traffic-related noise/pollution and dementia incidence could not be explained by SES.
Chen et al., 2017 [70]	Canada	Longitudinal	2066,639 adults (55–85 years old)	Exposure: Air pollution (NO_2_, PM_2.5_, O_3_) Covariates: Income, education, region, medical history, etc.	Dementia incidence	People from the lower and lower-middle income groups made up more of the dementia cases than the middle- and upper-income groups; Dementia incidence was not associated with educational attainment; Adjusting for income and education attenuated the association between PM_2.5_ and NO_2_ exposures and dementia incidence; No association was found between O_3_ and dementia.
Evangelinakis et al., 2024 [58]	Multiple	Review	Women	Exposure: Endocrine-disrupting chemicals (BPA, PCB, phthalates) Covariates: Age, socioeconomic status	Premature ovarian aging	Endocrine-disrupting chemical exposure was on average higher in women with low socioeconomic status; Literature supports possible links between endocrine-disrupting chemicals and premature ovarian insufficiency, menopause, and infertility. Epigenetic regulation and oxidative stress may be involved.
Gatto et al., 2014 [71]	USA	Cross-sectional	1496 adults	Exposure: Air pollution (NO_2_, PM_2.5_, O_3_) Covariates: Education, income, sex, age, race	Cognitive function	Participants with lower income and less education were more exposed to NO_2_ and PM_2.5_; An increase in PM_2.5_ exposure was associated with lower verbal learning, NO_2_ exposure was associated with lower logical memory, and O_3_ exposure was associated with lower executive function.
Gerber et al., 2014 [34]	Israel	Longitudinal	1120 adults aged 65+	Exposure: PM_2.5_ Effect modifier: Frailty Covariate: Socioeconomic status	Age-related Post-Myocardial Infarction Mortality	Participants loss to follow-up were more likely to be older, frailer, female, and presented with a poorer socioeconomic status; PM_2.5_ exposure was associated with increased odds of developing frailty post-myocardial infarction.
Keidel et al., 2019 [24]	Multiple (Europe)	Cross-sectional	6502 adults	Exposure: Traffic-related air pollution (NO_2_ exposure based on place of residence) Covariates: Socioeconomic status	Lung function (FEV1, FEV)	High-educated participants were more exposed to NO_2_ and low-educated individuals had the lowest lung function; Education played a role in the relationship between pollution and lung function decline; Results indicate that the inverse association between NO_2_ and lung function held even when adjusting for socioeconomic status.
Leng et al., 2022 [72]	USA	Longitudinal	2511 adults (40–75 years old)	Exposures: Wood smoke Covariate: Socioeconomic Status	Lung aging, health-related quality of life, and mortality	29% reported being exposed to wood smoke over a year, with exposed individuals experiencing quicker lung function decline than unexposed participants; Wood smoke exposure shortened life span, with most being younger, Hispanic, lower income and less educated
Massa et al., 2016 [73]	Brazil	Cross-sectional	1333 adults aged 60+	Exposures: Green space, income inequality, and education Covariates: Smoking status, alcohol intake, BMI	Cardiovascular disease (CVD)	Higher educational attainment was associated with lower CVD prevalence; Middle-income participants with good access to green spaces had lower odds of CVD (OR = 0.44, 95% CI: 0.39–0.49) compared to those without green space access (OR = 1.35, 95% CI: 1.15–1.59).
McGuinn et al., 2016 [74]	USA	Longitudinal	9334 adults	Exposure: PM_2.5_ Covariates: Education, gender, race, smoking status	Coronary Artery Disease	A 1 μg/m^3^ increase in the annual average of PM_2.5_ was associated with an 11.1% relative increase in the odds of coronary artery disease, adjusting for education, gender, race, and smoking.
Ruiz et al., 2018 [63]	Multiple	Review	Adults	Exposure: Environmental exposures (BPA, phthalates, air pollution) Covariates: Household income, race	Diabetes risk and outcomes	Non-white individuals with lower household income are more likely to be diabetic; Diabetogenic pollutant exposure was associated with increased diabetes risk and worse outcomes among those with lower income.
Trevenen et al., 2022 [37]	Australia	Longitudinal	11,243 men aged 65+	Exposure: Low levels of air pollution (NO_2_, PM_2.5_, black carbon) Covariates: Socioeconomic status, education	Dementia incidence (Alzheimer’s disease, vascular dementia)	PM_2.5_ was associated with increased vascular dementia incidence before adjusting for socioeconomic status. This association was attenuated once adjusting for such conditions.
White et al., 2019 [75]	USA	Cross-sectional	2878 women (35–74 years old)	Exposure: Air pollution (NO_2_, PM_10_, PM_2.5_) Covariates: Socioeconomic status	Epigenetic age acceleration	An increase in NO_2_ was inversely associated with age acceleration (β = −0.24, 95% CI: −0.47, −0.02); PM_2.5_ and PM_10_ were not significantly associated with age acceleration; Adjusting for socioeconomic status did not notably change results.
Yamamoto et al., 2019 [56]	Multiple (China, Russia, Ghana, India, Mexico, South Africa)	Cross-sectional	Adults (50+ years old)	Exposure: Household pollution (type of fuel: electricity, gas, wood, coal, kerosene, agriculture, etc.) Covariates: Socio-demographics, education, and household income	Arthritis	The distribution of arthritis was similar across income groups; University/College graduates had lower odds of arthritis (aOR = 0.45, *p* < 0.001) than those with primary education; Highest odds of arthritis were observed for those who use agriculture (crops, grass, etc.) as fuel (aOR = 0.94, *p* = 0.83).
Yoon et al., 2023 [76]	South Korea	Cross-sectional	1190 adults (60–98 years old)	Exposure: Phthalates Covariates: Socio-demographics, income, education, housing, etc.	Walking speed	Slower walking participants were significantly older and had lower education, weight, and exercise levels; Higher levels of phthalate metabolite mixtures found in urine were linked to slower walking speeds.
Section B: Association between environmental factors and aging outcomes—modifying effect of socioeconomic conditions						
Cui et al., 2024 [32]	China	Cross-sectional	108,941 adults	Exposure: Air pollution (PM_2.5_, ammonium, black carbon, nitrates, organic matter, sulfates) Effect modifier: Socioeconomic status	Cardiometabolic multi-morbidity	Air pollution exposure was higher among farmers and the low-income group; The relationship between the air pollutants and cardiometabolic multi-morbidity varied by socioeconomic group; Lower socioeconomic status exacerbated health risks associated with PM_2.5_.
Heo et al., 2022 [77]	South Korea	Longitudinal	84,544 adults aged 50+	Exposure: Air pollution (PM_10_, SO_2_, CO, NO_2_, and O_3_) Effect modifiers: Sex, age, exercise level, income	Risk of osteoporosis-related fracture	Associations between air pollution exposure and fractures were weak, with the only marginally significant hazard ratio being for SO_2_ exposure (HR = 1.04; 95% CI: 1.0, 1.08); Air pollution levels varied across income groups. There was no evidence of effect modification.
Jones et al., 2020 [78]	USA	Longitudinal (Case-crossover)	5336 adults	Exposure: Wildfire-related particulate matter Effect modifiers: Socioeconomic status, age, sex	Cardiac arrests	Both high and low socioeconomic groups had elevated odds ratios when exposed to heavy smoke When exposed to light and medium smoke levels, only the low socioeconomic strata had elevated cardiac arrest prevalence.
Kim et al., 2023 [79]	USA	Longitudinal	924 adults	Exposure: Green space Effect modifier: Neighborhood socioeconomic status	Epigenetic aging	Greater greenness was associated with slower epigenetic aging; The association between greenness and epigenetic aging was stronger among participants in disadvantaged neighborhoods.
Lee et al., 2023 [80]	USA	Longitudinal	7056 adults	Exposure: PM_2.5_ Effect modifier: Neighborhood socioeconomic disadvantage Covariates: Employment status, family income, race, sex, age, smoking status	Self-rated health	PM_2.5_ exposure was inversely associated with self-rated health, which was observed across all socioeconomic groups; In disadvantaged neighborhoods, this association was exacerbated (more negative), whereas the harmful impact of PM_2.5_ on self-rated health was weaker in the context of greater neighborhood disadvantage.
Qiu et al., 2022 [81]	USA	Longitudinal	570 adults	Exposure: Air pollution (O_3_, PM_2.5_, NO_2_) Effect modifier: Area-level income Covariates: Household income, education, marital status, age, sex, BMI	Aging-related psychiatric symptoms	Positive association between ambient pollutant exposures (NO_2_ and O_3_) and elevated psychiatric symptom intensity; No association between PM_2.5_ and psychiatric symptoms; Area-level income and house value modified the association between O_3_ exposure and psychiatric symptoms, the same was not observed for other pollutants.
Triebner, et al., 2019 [23]	Multiple (Europe)	Longitudinal	1955 women	Exposure: Green space Effect modifiers: Education, age at completed education	Age at menopause	Women with access to little green space became menopausal 1.4 years earlier than those with good access to green spaces; High educational attainment was associated with an older age at menopause. However, it did not modify the relationship between green space access and age at menopause; The impact of green space accessibility was strongest in Northern Europe and becomes weaker in Central Europe and attenuates in Southern Europe.
Wang et al., 2022 [82]	China	Longitudinal	1286 adults	Exposure: Neighborhood environment (urban density, walkability) Effect modifier: Education	Cognitive decline related to aging	The protective effect of higher educational attainment in the slowing of cognitive decline was exacerbated in participants living in disadvantaged rural neighborhoods.
Section C: Association between socioeconomic conditions and aging outcomes—modifying effect of environmental factors						
Koh et al., 2022 [26]	USA	Cross-sectional	3887 adults	Exposure: Socioeconomic disparities (income, education) Effect modifiers: Green space access	Hypertension	A dose-response relationship was found between educational attainment and hypertension; Green space accessibility did not modify the relationship.
Mitchell et al., 2015 [51]	Multiple (Europe)	Cross-sectional	21,294 adults	Exposure: Financial strain Effect modifiers: Neighborhood (green spaces, accessibility, etc.)	Mental and cognitive well-being	Green space access had a significant interaction in the association between financial strain and mental well-being; Socioeconomic inequality in mental well-being was 40% narrower among those with access to green spaces, compared to those with poorer access.
Vilarino-Rico et al., 2023 [83]	Spain	Longitudinal (retrospective follow-up)	770 adults	Exposure: Personal and household income Effect modifiers: Population density	Major Adverse Cardiovascular Events (MACEs)	Living in a thinly populated area was associated with lower risk of MACE; Income not associated with worse outcomes.
Section D: Association between socioeconomic conditions and aging outcomes—mediating effect of environmental factors						
Albers et al., 2024 [16]	Netherlands	Cross-sectional	9188 adults	Exposure: Socioeconomic position (education, income, occupation) Mediators: Green spaces, walkability	Type 2 diabetes	Lower socioeconomic position was associated with higher incidence of type 2 diabetes, less healthy food options, less green space access, and less walkable spaces; The association between socioeconomic position and type 2 diabetes was not strongly mediated by environment (0.1%).
Canterbury et al., 2020 [84]	USA	Longitudinal	1933 adults (45–75 years old)	Exposure: Social risk (racial minority, single living, low income, low educational status) Mediators: PM_2.5_	Cardio- vascular disease (CVD) risk	Increased social risk was associated with higher CVD risk; 13% of the association between social risk and CVD risk was explained by PM_2.5_ exposure.
Chaparro et al., 2018 [85]	United Kingdom	Longitudinal	85,875 adults	Exposure: Socioeconomic deprivation Mediators: SO_2_, PM_10_, NO_2_, CO	Forced expiratory volume in 1s (FEV1%), systolic blood pressure, BMI, and levels of C-reactive protein	Only SO_2_ partially mediated the positive association between socioeconomic deprivation and systolic blood pressure, BMI, and C-reactive protein, with FEV1% not being associated.
Quispe-Haro et al., 2024 [25]	Czech Republic	Cross-sectional	6381 adults	Exposure: Education Mediators: Air pollution (NO_2_ and PM_10_)	Lung function (FEV1, FEV)	Higher levels of education were associated with lower exposures to pollution; Individuals with higher education had better lung function than those with only primary education; Roughly 12% of association between education and lung function was mediated by air pollution exposure (NO_2_ and PM_10_).

## Data Availability

No new data were created or analyzed in this study.

## Abbreviations

The following abbreviations are used in this manuscript:

PM_2.5_	Fine particulate matter
PM_10_	Ultra-fine particulate matter
SO_2_	Sulfur dioxide
NO_2_	Nitrogen dioxide
O_3_	Ozone
BPA	Bisphenol A
FEV1	Forced expiratory volume in 1 s
CVD	Cardiovascular disease
MACE	Major Adverse Cardio-vascular Events
BMI	Body Mass Index

## Appendix A

(((Socioeconomic Factors/) OR (Social Class/) OR (Income) OR (Socioeconomic) OR (SES) OR (Social Determinants) OR (Poverty) OR (Education) OR (“Educational Attainment”) OR (“Food Security”) OR (Wealth) OR (Housing) OR (Occupation) OR (“Employment status”)) AND ((Pollut *) OR (Particulate matter) OR (“PM 2.5”) OR (“PM 10”) OR (“PM 0.1”) OR (Ozone) OR (“O3”) OR (Nitrogen Dioxide) OR (“NO2”) OR (Sulfur dioxide) OR (“SO2”) OR (Environment*) OR (Mold) OR (“Lead Poisoning”) OR (BPA) OR (bisphenol A) OR (Phthalates) OR (“Noise Pollution”) OR (“Water Quality”) OR (“Air Quality”) OR (“Green space”) OR (“Blue space”) OR (Walkab *)) AND ((Aging/) OR (Ag?ng) OR (Frail *) OR (Dementia/) OR (Alzheimer) OR (“Parkinson’s Disease”) OR (Bone Density/) OR (“Lung function”) OR (“Cognitive Function”) OR (Memory) OR (Respirat*) OR (Cancer/) OR (Vascular) OR (COPD) OR (Cardiovascular) OR (Hypertension) OR (Chronic) OR (“Walking Speed”) OR (Balance) OR (“Epigenetic Age”) OR (“Biological age”) OR (Longevity) OR (Mortality) OR (Life expectancy) OR (“Grip strength”) OR (“Age-related”) OR (Function)))
